# Supplementary material for: Therapeutic role of miR-19a/19b in cardiac regeneration and protection from myocardial infarction
Source: Nat Commun. 2019 Apr 17;10:1802. doi: 10.1038/s41467-019-09530-1 (PMC6470165; doi:10.1038/s41467-019-09530-1)

Gao et al., Therapeutic role of miR-19a/19b in cardiac regeneration and protection from myocardial infarction

Supplementary Information

Supplementary Table1. Echocardiography examination of cardiac function after intra-cardiac injection of individual miR-19a, miR-19b or control mimics at multiple time points post-MI.

	MI-5d			MI-2w			MI-7w			MI-9w		
	Control (N=5)	miR-19a (N=4)	miR-19b (N=5)	Control (N=5)	miR-19a (N=4)	miR-19b (N=6)	Control (N=4)	miR-19a (N=3)	miR-19b (N=5)	Control (N=4)	miR-19a (N=4)	miR-19b (N=5)
IVS;d(mm)	0.623±0.028	1.014±0.049**	1.368±0.043***	0.750±0.064	0.869±0.040	1.213±0.015**	0.976±0.047	0.841±0.051	0.971±0.016	0.747±0.018	0.850±0.037	0.992±0.029*
IVS;s(mm)	0.795±0.024	1.532±0.033**	1.654±0.056***	0.999±0.010	1.417±0.039	1.749±0.014**	1.513±0.036	1.417±0.080	1.352±0.056	1.101±0.087	1.304±0.028	1.422±0.033
LVID;d(mm)	3.665±0.107	3.468±0.075	3.263±0.101	3.905±0.081	3.268±0.106	3.383±0.078*	3.142±0.099	3.022±0.237	3.745±0.028**	3.989±0.100	3.915±0.112	4.057±0.149
LVID;s(mm)	3.183±0.137	2.231±0.138*	2.452±0.094*	3.220±0.113	2.170±0.148*	2.223±0.116*	2.273±0.095	1.777±0.221	2.552±0.051	3.171±0.116	2.608±0.167	2.782±0.149
LVPW;d(mm)	0.716±0.045	0.876±0.040	1.033±0.020*	0.891±0.009	0.953±0.027	0.865±0.055	0.905±0.044	0.987±0.010	0.909±0.012	0.921±0.019	0.892±0.024	0.893±0.029
LVPW;s(mm)	0.801±0.047	1.294±0.080*	1.370±0.043**	1.151±0.034	1.307±0.049	1.333±0.078	1.378±0.057	1.503±0.037	1.450±0.030	1.207±0.071	1.394±0.049	1.393±0.084
EF(%)	29.765±2.904	64.177±5.301*	48.95±3.438*	37.160±3.069	63.226±4.050*	63.540±2.986*	54.894±2.269	74.268±3.405*	60.523±1.405	41.954±3.202	62.757±3.503*	60.370±2.055*
FS(%)	13.905±1.492	35.838±3.617*	28.247±1.529*	17.991±1.657	34.335±2.887*	32.680±4.544*	27.775±1.496	42.307±2.661*	31.988±0.980	20.586±1.834	34.190±2.518*	32.164±1.407*
LV Mass(mg)	81.080±5.915	118.765±5.158*	156.161±9.908**	116.427±3.932	104.325±7.972	135.851±12.487	100.676±4.753	93.367±12.100	132.468±2.728*	123.697±3.46	130.690±10.396	148.531±3.222*
LV Mass(Corrected,mg)	64.863±4.732	95.012±4.127*	124.929±7.927**	93.142±3.146	83.460±6.377	108.681±9.990	80.450±3.802	74.693±9.680	105.974±2.183*	98.958±2.770	104.552±8.317	118.825±2.577*
LV Vol;d(ul)	58.328±3.744	50.175±2.666	47.128±3.658	66.966±3.100	43.986±3.598*	51.564±4.919*	39.946±3.158	37.800±7.069	59.984±1.028**	70.348±4.136	67.514±4.526	75.325±6.465
LV Vol;s(ul)	42.842±3.900	18.162±2.681*	21.708±2.398*	43.208±3.196	17.223±2.705	21.711±4.374*	18.251±1.994	11.085±3.430	23.970±0.141	41.111±3.563	26.881±3.886	31.888±4.034

Wild type C57BL/6 mice were randomly subjected to intra-cardiac injection of miR-19a, miR-19b or control mimics after myocardium infarction (MI) surgery, respectively. Echocardiography analyses of cardiac function were performed at multiple time points post-MI surgery from miR-19a/19b and control group. N of each group was indicated. *: *P<0.05*; **: *P<0.01* vs. control group. IVS;d: Interventricular septal thickness at diastole; IVS;s Interventricular septal thickness at systole; LVID;d: Left ventricular end diastolic internal dimension; LVID;s: Left ventricular end systolic internal dimension; LVPW;d: Left ventricular end diastolic posterior wall dimension; LVPW;s: Left ventricular end systolic posterior wall dimension; EF: Ejection fraction; FS: Fractional shortening; LV Vol;d: Left ventricular end diastolic volume. LV Vol;s: Left ventricular end systolic volume.

Supplementary Table2. Echocardiography examination of cardiac function after intra-cardiac injection of miR-19a/19b or control mimics at multiple time points of post-MI.

	**2 - 4 weeks post-MI**		**2 months post-MI**		**4 months post-MI**		**1 year post-MI**	
	**Control (n=8)**	**miR-19a/19b (n=12)**	**Control (N=5)**	**miR-19a/19b (N=10)**	**Control (N=5)**	**miR-19a/19b (N=10)**	**Control (N=3)**	**miR-19a/19b (N=4)**
**IVS;d (mm)**	0.463±0.074	0.523±0.093	0.735±0.219	0.606±0.088	0.921±0.160	0.731±0.093*	0.561±0.134	1.036±0.229*
**IVS;s (mm)**	0.520±0.074	0.634±0.083**	0.767±0.238	0.861±0.148	1.151±0.162	0.977±0.088*	0.604±0.178	1.362±0.209**
**LVID;d (mm)**	5.024±0.805	4.383±0.522*	5.693±0.707	4.773±0.601*	5.733±1.322	4.937±0.524	5.299±0.818	4.680±0.632
**LVID;s (mm)**	4.038±0.948	3.027±0.550**	4.824±0.921	3.464±0.723**	4.970±1.503	3.641±0.686*	5.010±0.945	3.762±0.619
**LVPW;d (mm)**	0.483±0.043	0.575±0.029**	0.730±0.198	0.703±0.077	0.877±0.141	0.787±0.105	1.285±0.251	1.069±0.270
**LVPW;s (mm)**	0.637±0.080	0.774±0.110**	0.848±0.180	0.947±0.112	1.010±0.082	0.980±0.179	1.525±0.265	1.313±0.195
**EF (%)**	41.05±12.16	59.04±8.20**	32.51±11.06	53.72±10.45**	29.62±11.39	51.51±11.32**	12.853±7.755	39.24±14.61*
**FS (%)**	20.43±6.90	31.34±5.55**	15.78±5.79	28.08±6.76**	14.20±5.69	26.79±7.34**	5.786±3.560	19.48±8.46*
**LV Mass (mg)**	90.77±20.68	85.50±19.12	189.06±45.08	124.86±36.07*	257.47±102.49	159.40±39.33*	238.43±116.70	218.36±34.36
**LV Mass (Corrected, mg)**	72.62±16.55	68.40±15.29	151.25±36.06	99.89±28.86*	205.98±81.99	127.52±31.47*	190.74±93.36	174.69±27.48
**LV Vol;d (uL)**	123.39±45.51	88.58±24.58*	162.33±47.56	108.30±31.62*	171.78±100.73	116.48±27.10	138.30±49.93	103.38±29.45
**LV Vol;s (uL)**	76.79±41.78	37.56±16.16**	113.36±52.01	52.67±25.59**	128.91±101.62	58.67±24.10	122.80±53.69	62.50±24.55
**Heart Rate (BMP)**	626±31	633±24	612±66	665±21*	585±60	631±16*	533.04±42.55	478.68±87.03

Wild type C57BL/6 mice were randomly subjected to intra-cardiac injection of miR-19a/19b or control mimics after myocardium infarction (MI) surgery, respectively. Echocardiography analyses of cardiac function were performed at multiple time points post-MI surgery from miR-19a/19b and control group. N of each group was indicated. *: *P<0.05*; **: *P<0.01* vs. control group. IVS;d: Interventricular septal thickness at diastole; IVS;s Interventricular septal thickness at systole; LVID;d: Left ventricular end diastolic internal dimension; LVID;s: Left ventricular end systolic internal dimension; LVPW;d: Left ventricular end diastolic posterior wall dimension; LVPW;s: Left ventricular end systolic posterior wall dimension; EF: Ejection fraction; FS: Fractional shortening; LV Vol;d: Left ventricular end diastolic volume. LV Vol;s: Left ventricular end systolic volume.

Supplementary Table 3. Echocardiography examination of cardiac function after intra-cardiac injection of AAV9-cTNT-miR-19a/19b or control AAV at multiple time points post-MI.

	1 day post-MI		7 days post-MI		2 weeks post-MI		2 months post-MI		3 months post-MI	
	AAV-Ctrl (n=4)	AAV-miR-19a/b (n=6)	AAV-Ctrl (n=4)	AAV-miR-19/b (n=6)	AAV-Ctrl (n=4)	AAV-miR-19a/b (n=6)	AAV-Ctrl (n=6)	AAV-miR-19a/b (n=6)	AAV-Ctrl (n=6)	AAV-miR-19a/b (n=7)
IVS;d (mm)	.832±0.117	0.994±0.109	0.653±0.184	0.842±0.187	0.563±0.086	0.577±0.022	0.718±0.123	0.622±0.084	0.595±0.040	0.583±0.078
IVS;s (mm)	1.481±0.172	1.509±0.096	0.912±0.346	1.313±0.277*	0.617±0.102	0.657±0.103	0.753±0.129	0.823±0.088	0.644±0.149	0.747±0.083
LVID;d (mm)	3.261±0.727	3.245±0.238	4.087±0.389	3.905±0.391	4.554±0.466	3.754±0.647	5.024±0.603	4.387±0.568	5.082±0.402	4.273±0.483**
LVID;s (mm)	1.991±0.728	2.128±0.325	3.338±0.659	2.846±0.417	3.595±0.628	2.291±0.537**	4.190±1.015	2.804±0.502*	4.218±0.646	2.833±0.660**
LVPW;d (mm)	0.981±0.494	0.855±0.139	0.537±0.129	0.510±0.092	0.667±0.107	0.688±0.079	0.736±0.139	0.675±0.047	0.610±0.073	0.701±0.084
LVPW;s (mm)	1.373±0.449	1.193±0.226	0.748±0.166	0.788±0.188	0.771±0.120	0.849±0.150	0.805±0.104	0.932±0.143	0.687±0.117	0.882±0.067**
EF (%)	71.276±13.160	64.015±11.407	38.542±16.902	53.474±8.388	43.32±10.18	70.27±6.86**	35.11±19.84	66.28±5.77**	35.62±12.48	62.63±12.59**
FS (%)	40.754±11.513	34.466±8.696	19.019±9.830	27.324±5.320	21.45±5.63	39.34±5.83**	17.48±10.47	36.49±4.29**	17.42±6.92	34.32±9.49**
LV Mass (mg)	97.872±14.605	102.926±8.454	82.520±14.168	90.889±19.562	104.03±16.21	79.39±26.37	155.65±43.46	104.61±18.61*	123.54±14.83	100.02±25.33
LV Mass (Corrected, mg)	78.297±11.684	82.341±6.763	66.016±11.334	72.711±15.650	83.23±12.97	63.51±21.10	124.52±34.76	83.69±14.89*	98.83±11.86	80.02±20.27
LV Vol;d (uL)	45.592±21.514	42.679±7.496	74.494±15.712	66.942±15.132	96.18±24.05	62.47±25.26	121.60±34.32	88.85±23.70	123.73±21.45	83.16±21.88**
LV Vol;s (uL)	15.001±10.537	15.390±5.537	47.685±19.217	31.679±10.651	56.20±24.89	19.40±11.21*	83.86±49.05	30.95±11.10*	81.67±26.79	32.77±17.26**

Wild type C57BL/6 mice were randomly subjected to intra-cardiac injection of AAV-miR-19a/19b or control AAV after myocardium infarction (MI) surgery, respectively. Echocardiography analyses of cardiac function were performed at multiple time points post-MI surgery from miR-19a/19b and control group. N of each group was indicated. *: *P<0.05*; **: *P<0.01* vs. control group. IVS;d: Interventricular septal thickness at diastole; IVS;s Interventricular septal thickness at systole; LVID;d: Left ventricular end diastolic internal dimension; LVID;s: Left ventricular end systolic internal dimension; LVPW;d: Left ventricular end diastolic posterior wall dimension; LVPW;s: Left ventricular end systolic posterior wall dimension; EF: Ejection fraction; FS: Fractional shortening; LV Vol;d: Left ventricular end diastolic volume. LV Vol;s: Left ventricular end systolic volume.

Supplementary Table 4. Echocardiography examination of cardiac function after tail-vein injection of LANCER II delivered miR-19a/19b in adult mice.

	MI-2w		MI-6w		MI-11w		MI-14w	
	Control (N=4)	miR-19a/19b (N=4)	Control (N=4)	miR-19a/19b (N=4)	Control (N=5)	miR-19a/19b (N=10)	Control (N=5)	miR-19a/19b (N=6)
IVS;d(mm)	0.905±0.023	1.127±0.033*	0.689±0.168	1.088±0.019*	0.693±0.044	1.047±0.017**	0.804±0.068	0.970±0.022
IVS;s(mm)	1.236±0.037	1.755±0.043**	1.191±0.278	1.697±0.029	1.115±0.068	1.546±0.027**	1.216±0.112	1.520±0.024
LVID;d(mm)	3.538±0.097	2.504±0.458**	3.683±0.146	3.374±0.114	3.861±0.159	3.570±0.087	4.217±0.221	3.812±0.086
LVID;s(mm)	2.669±0.084	1.581±0.106**	2.473±0.036	2.022±0.104	2.926±0.222	2.379±0.104	3.459±0.285	2.398±0.082
LVPW;d(mm)	1.046±0.096	1.747±0.046**	0.766±0.059	0.860±0.025	0.943±0.037	0.992±0.029	0.909±0.037	0.884±0.012
LVPW;s(mm)	1.391±0.135	2.002±0.032*	1.320±0.059	1.423±0.014	1.334±0.079	1.547±0.032	1.149±0.048	1.472±0.021
EF(%)	49.449±2.103	68.663±2.688*	61.593±1.062	71.961±1.690*	40.386±2.560	63.489±2.044*	40.229±4.795	67.588±1.378*
FS(%)	24.560±1.233	37.254±2.172*	32.501±0.800	40.398±1.398*	19.671±1.425	36.113±1.597*	20.433±2.715	37.431±1.220*
LV Mass(mg)	128.490±8.735	149.726±9.956	84.608±13.046	119.694±6.832	126.771±6.592	137.607±3.990	134.345±5.245	135.573±5.928
LV Mass(Corrected,mg)	102.792±6.988	119.781±7.964	70.369±9.782	95.755±5.465	101.418±5.274	110.086±3.192	107.476±4.196	108.459±4.742
LV Vol;d(ul)	52.969±3.513	23.392±2.726**	57.618±2.570	47.579±3.829	72.306±5.481	57.865±3.454	85.816±10.287	63.903±3.330
LV Vol;s(ul)	42.842±3.900	7.698±1.357**	21.589±0.385	10.750±4.396	36.466±4.444	12.106±1.296**	44.631±9.1300.97	19.163±1.776

Wild type C57BL/6 mice were randomly subjected to tail-vein injection of miR-19a/19b or control mimics after myocardium infarction (MI) surgery, respectively. Echocardiography analyses of cardiac function were performed at multiple time points post-MI surgery from miR-19a/19b and control group. N of each group was indicated. *: *P<0.05*; **: *P<0.01* vs. control group. IVS;d: Interventricular septal thickness at diastole; IVS;s Interventricular septal thickness at systole; LVID;d: Left ventricular end diastolic internal dimension; LVID;s: Left ventricular end systolic internal dimension; LVPW;d: Left ventricular end diastolic posterior wall dimension; LVPW;s: Left ventricular end systolic posterior wall dimension; EF: Ejection fraction; FS: Fractional shortening; LV Vol;d: Left ventricular end diastolic volume. LV Vol;s: Left ventricular end systolic volume.

Supplementary Table 5. Echocardiography examination of cardiac function after tail-vein injection of RNAiMAX delivered miR-19a/19b in adult mice.

	MI-2w		MI-6w		MI-11w		MI-14w	
	Control (N=7)	miR-19a/19b (N=7)	Control (N=7)	miR-19a/19b (N=7)	Control (N=9)	miR-19a/19b (N=9)	Control (N=8)	miR-19a/19b (N=8)
IVS;d(mm)	1.047±0.013	0.990±0.015	0.648±0.026	0.920±0.019**	0.794±0.016	1.083±0.026**	0.629±0.028	0.892±0.028*
IVS;s(mm)	1.522±0.015	1.612±0.020	0.889±0.048	1.459±0.025***	1.032±0.036	1.545±0.020***	0.794±0.044	1.357±0.043**
LVID;d(mm)	3.424±0.090	3.006±0.088	4.428±0.079	3.245±0.066***	4.146±0.067	3.190±0.051***	4.635±0.045	3.588±0.057***
LVID;s(mm)	2.375±0.099	1.577±0.056*	3.567±0.107	1.939±0.078***	3.324±0.094	2.046±0.045***	3.973±0.073	2.297±0.047***
LVPW;d(mm)	0.850±0.015	0.962±0.034	0.784±0.015	0.951±0.021*	0.778±0.021	0.913±0.028	0.778±0.017	0.912±0.017*
LVPW;s(mm)	1.242±0.017	1.507±0.022**	1.194±0.022	1.549±0.027**	1.136±0.032	1.420±0.025*	1.019±0.025	1.386±0.021***
EF(%)	59.618±1.985	80.252±0.795**	40.088±2.215	72.061±1.716***	37.025±1.410	66.626±0.930***	30.463±1.638	65.919±1.206***
FS(%)	31.551±1.319	47.795±0.821***	19.915±1.293	41.143±1.422***	17.904±0.732	36.138±0.741***	14.595±0.904	36.014±0.920***
LV Mass(mg)	118.148±4.615	100.363±3.583	120.366±3.335	105.834±3.093	124.260±3.641	114.742±3.576	128.396±3.952	117.761±3.537
LV Mass(Corrected,mg)	94.518±3.692	80.291±2.866	96.293±2.668	84.668±2.474	99.408±2.913	91.793±2.861	102.717±3.161	94.209±2.830
LV Vol;d(ul)	50.461±3.003	37.238±2.516	90.772±3.725	43.572±2.062***	81.362±2.730	41.848±1.580***	99.819±2.276	55.200±2.095***
LV Vol;s(ul)	22.158±2.139	7.627±0.639*	47.524±2.585	13.239±1.205	52.420±2.611	14.322±0.699***	70.842±2.990	18.824±0.864***

Wild type C57BL/6 mice were randomly subjected to tail-vein injection of miR-19a/19b or control mimics after myocardium infarction (MI) surgery, respectively. Echocardiography analyses of cardiac function were performed at multiple time points post-MI surgery from miR-19a/19b and control group. N of each group was indicated. *: *P<0.05*; **: *P<0.01* vs. control group. IVS;d: Interventricular septal thickness at diastole; IVS;s Interventricular septal thickness at systole; LVID;d: Left ventricular end diastolic internal dimension; LVID;s: Left ventricular end systolic internal dimension; LVPW;d: Left ventricular end diastolic posterior wall dimension; LVPW;s: Left ventricular end systolic posterior wall dimension; EF: Ejection fraction; FS: Fractional shortening; LV Vol;d: Left ventricular end diastolic volume. LV Vol;s: Left ventricular end systolic volume.

Supplementary table 6. Antibodies used in this study

**Primary antibodies**				
**Antigen**	**Company**	**Catalog #**	**Origin**	**Working dilution**
Socs1	Abcam	ab9870	Goat	1:1000 for WB
Socs3	Abcam	ab16030	Rabbit	1:1000 for WB
STAT3	CST	9139	Mouse	1:1000 for WB
p-STAT3	CST	9145	Rabbit	1:1000 for WB
PTEN	CST	9188	Rabbit	1:1000 for WB
Bim	CST	2933	Rabbit	1:1000 for WB
c-caspase3	CST	9661	Rabbit	1:1000 for WB
Bcl2	CST	3498	Rabbit	1:1000 for WB
Bax	CST	2772	Rabbit	1:1000 for WB
CD80	Hangzhou Huaan Biotechnology	M1007-10	Mouse	1:50 for IHC
iNOS	Hangzhou Huaan Biotechnology	RT1332	Rabbit	1:200 for IHC
Arginase1	Hangzhou Huaan Biotechnology	RT1051	Rabbit	1:200 for IHC
phospho-Histone H3	Millipore	06-570	Rabbit	1:400 for IF
Aurora B	Abcam	ab2254	Rabbit	1:100 for IF
Connexin 43	Abcam	ab11370	Rabbit	1:1000 for IF
PAN cadherin	Sigma	C3678	Rabbit	1:500 for IF
Alpha-actinin	Abcam	ab9465	Mouse	1:250 for IF
cTNT	DSHB	CT3	Mouse	1:500 for IF
**Secondary antibodies**				
Alexa Fluor® 594 Goat Anti-Mouse	Invitrogen	A-11005	Goat	1:400 for IF
Alexa Fluor® 594 Goat Anti-Rabbit	Invitrogen	A-11012	Goat	1:400 for IF
Alexa Fluor® 488 Goat Anti-Mouse	Invitrogen	A-11001	Goat	1:400 for IF
Alexa Fluor® 488 Goat Anti-Rabbit	Invitrogen	A-11008	Goat	1:400 for IF

Supplementary table 7. Primers Used in This Study

**SYBR Green PCR primers**		
Gene	Forward	Reverse
Mst1	GCCTCCAGCTTAGGAGAATG	GGAAGTCATTCAGTGGTGAGC
Mst2	TGCTGATATACATCCGATGAGG	CAGACCAAAGTTCAGGTTTCCT
Lats1	TCCACAGATGTTTCAGGATTTG	GAAGAGCTTGAATAACCATGTCC
Lats2	GAGGTGCTTCTCCGCAAA	AGCATCTCAAAGAGAATCACACC
Mob1b	AACCTACGGATGGCTGTCA	AAAATCCACAGCTGCAAAGC
Yap	AAATGCTCCAAAATGTCAGGA	CATTCGGAGTCCCTCCATC
Taz	GCTGCAGACATCTGCTTCAC	TGAAGTCCATCCCTTTCTGG
Sav1	TACAGGCACCCCTGTGCT	TGTTGTGGCTGATACGTGATG
Irf9	TGAGGCCACCATTAGAGAGG	AGCAGCAGCGAGTAGTCTGA
PML	TGACCTCAAGATTGACAATGAAA	CAGGGAGACAGCTTTGGAGT
Sp100	AGGAATTGATGGAGGGAACC	AGCTGGCACCAGGTCCTT
Col2A1	GAAGGTGCTCAAGGTTCTCG	GCAATTCCAGGAGCACCA
Col4A1	TGGCACAAAAGGGACGAG	GGCCAGGAATACCAGGAAGT
Col8A1	GGCCAGCCAAGCCTAAAT	TGATGAACAGTATTCCCAGCA
Tgfb2	TTGTTCCACAGGGGTTAAGG	AGCTCGGTCCTTCAGATCCT
IFI35	TCACTTCCGAGTCAAGCTAGG	TTCTTGGTAGCATTAGGCAGTAAG
Bcl6	TTCCGCTACAAGGGCAAC	CAGCGATAGGGTTTCTCACC
Pik3ap1	GACGATGAGCTCTACGAGTCC	GGCCTGAAGCATTTCAACA
Xaf1	TGTTGTCAGTGCCGAATTTT	CCCACTGGAGTTTCTTTTGG
Slc15a2	TCTGCTATTTCACCCCCATC	CACCAGGGACAGATAGATGATTG
Acot1	TACGATGACCTCCCCAAGAA	CCAATTCCAGGTCCTTTTACC
Clec3b	GGAGAAGCAGGCCTTACAGA	ATGGAAGGTCTTCGGTTGG
Stmn2	GGAGCTGTCTATGCTGTCACTG	CTGCTTCACCTCCATGTCG
HDAC11	TGCAGACATCACACTGGCTAT	GGTGGGCATCGAGATCAA
IL-1-β	TCTTCCTAAAGTATGGGCTGGA	AAAGGGAGCTCCTTAACATGC
IL-6	CAAAGCCAGAGTCCTTCAGAG	GCCACTCCTTCTGTGACTCC
TNFα	CCACCACGCTCTTCTGTCTA	AGGGTCTGGGCCATAGAACT
CD68	CCAGCTGTTCACCTTGACCT	CAATGATGAGAGGCAGCAAG
MCP-1	GTTGGCTCAGCCAGATGCA	AGCCTACTCATTGGGATCATCTTG
Arginase 1	GAATCTGCATGGGCAACC	GAATCCTGGTACATCTGGGAAC
Cd163	TCACTTCTCAGTGCCTCTGC	CGCCAGTCTCAGTTCCTTCT
IL-10	ATCGATTTCTTCCCTGTGAA	TGGCCTTGTAGACACCTTGG
Socs1	GTGGTTGTGGAGGGTGAGAT	CCTGAGAGGTGGGATGAGG
Socs3	ATTTCGCTTCGGGACTAGC	AACTTGCTGTGGGTGACCAT
ANP	CAC AGA TCT GAT GGA TTT CAA GA	CCT CAT CTT CTA CCG GCA TC

BNP	GTC AGT CGT TTG GGC TGT AAC	AGA CCC AGG CAG AGT CAG AA
b-MHC	CGC ATC AAG GAG CTC ACC	CTG CAG CCG CAG TAG GTT
TGFBR2	GGCTCTGGTACTCTGGGAAA	AATGGGGGCTCGTAATCCT
ELN	TGGAGCAGGACTTGGAGGT	CCTCCAGCACCATACTTAGCA
FBN1	CCTTCCTGTGGCTCCAGAT	GCTGCCCCCATTCATACA
Bim	GGAGACGAGTTCAACGAAACTT	AACAGTTGTAAGATAACCATTTGAGG
Ctgf	TGACCTGGAGGAAAACATTAAGA	AGCCCTGTATGTCTTCACACTG
CDK1	CTCTATTAAAAGAACTTCGACATCCA	CCATGGACAGGAACTCAAAGA
Ccnb1	TGTGTGAACCAGAGGTGGAA	GGCTTGGAGAGGGATTATCA
Ccnd1	GAGATTGTGCCATCCATGC	CTCCTCTTCGCACTTCTGCT
THBS1	CACCTCTCCGGGTTACTGAG	GCAACAGGAACAGGACACCTA
**ABI Taqman probes accession number**		
TaqMan® MicroRNA Assays- hsa-miR-19a	4427975 (Assay ID 000395)	
TaqMan® MicroRNA Assays- hsa-miR-19b	4427975 (Assay ID 000396)	
TaqMan® MicroRNA Assays- U6 snRNA	4427975 (Assay ID 001973)	

Supplementary Data

Supplementary Figure1. Pharmacokinetics of miRNA mimics after intra-cardiac injection and myocardial infarction in adult mice.

Immediately after MI surgery, Cy-3 labeled micrONTM agomir Negative Control (10 μg per heart in 50 μl) were injected into 3 sites around the infarcted area (anterior wall, lateral wall, and apex area). At varied time points (2h, 6h, 12h, 24h, 48h) post myocardial infarction (MI), collected heart samples, performed immune-stating. Representative images of pharmacokinetics of miRNA mimics after intra-cardiac injection and MI in adult mouse heart. Cy3 marks miRNA mimic (red); α-actinin (ACTN1) marks cardiomyocytes (green); WGA marks cell surface (white) and DAPI labels nuclei (blue).

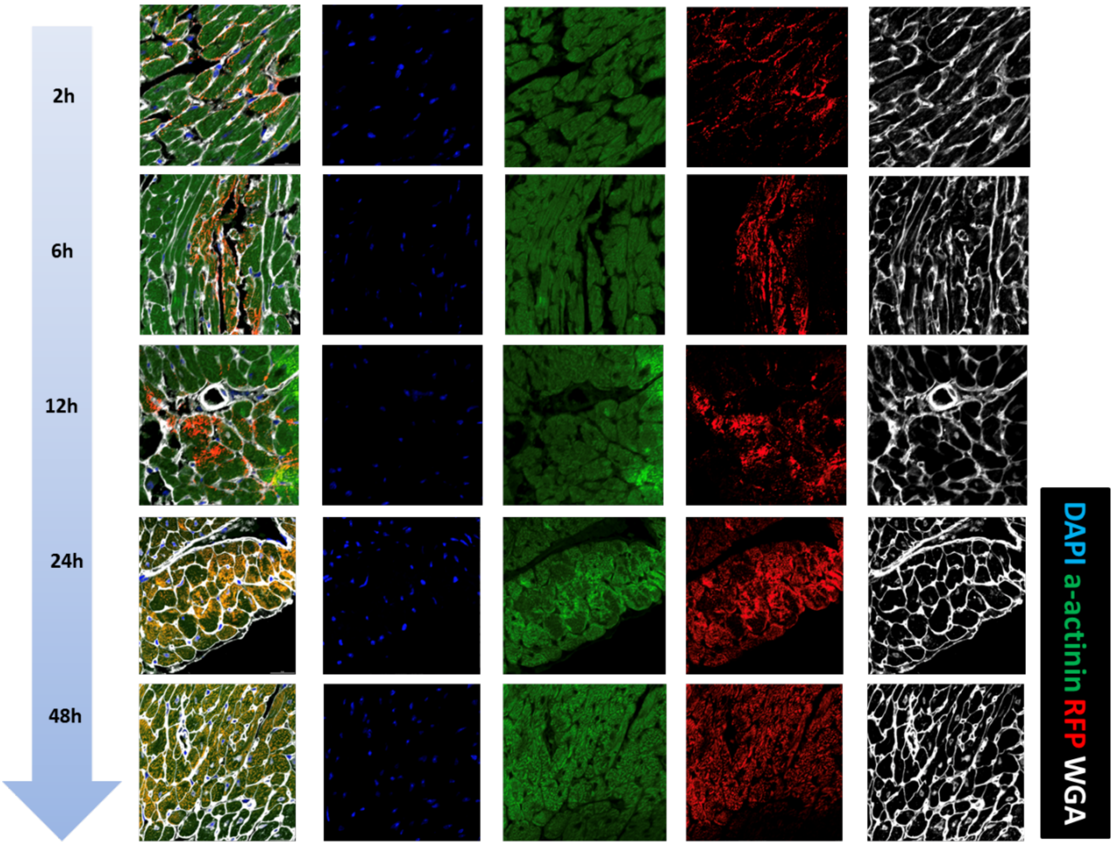

Supplementary Figure 2. Intra-cardiac injection of miR-19a/19b mimics in adult mice. (A) Kaplan-Meier survival curves of injection of miR-19a/19b mimics compared to injection of control mimic after MI injury. The n number for each group is indicated. (B) Triphenyltetrazolium chloride (TTC) staining of transverse section of hearts 24h after intra-cardiac injection of 19a/19b or control mimics post MI injury in adult mice. (C) Quantification of TTC staining. n=3. (D) Echocardiography analyses of left ventricle cardiac function of mice 24 h after intra-cardiac injection of individual miR-19a/19b or control mimics post MI. FS%, fractional shortening; EF%, ejection fraction.

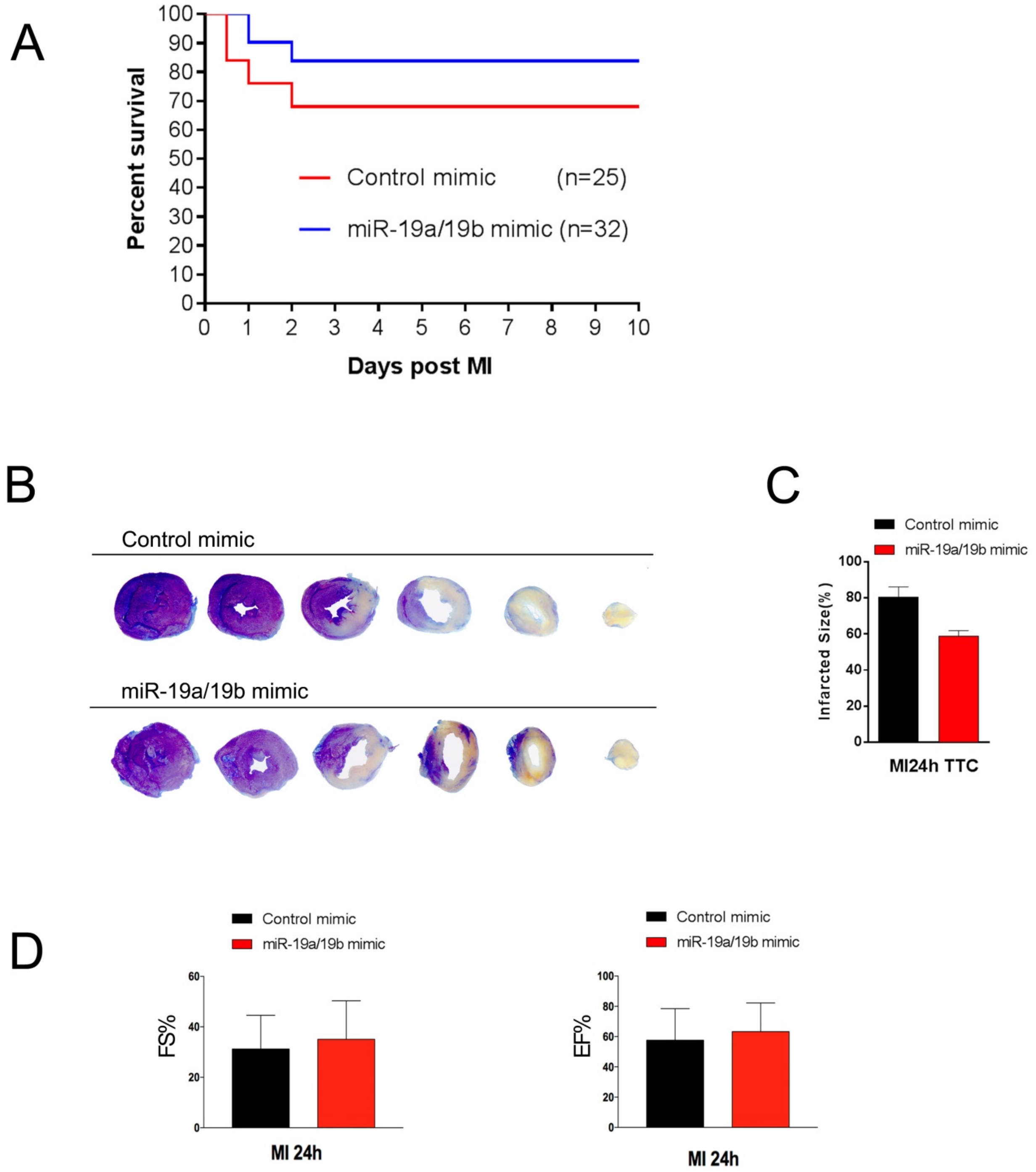

Supplementary Figure 3. Expression of Hippo/Yap pathway related gene. Quantitative RT-PCR (qPCR) analyses of the expression of Hippo/Yap pathway related genes (Yap, Taz, Tead1, Last1, Last2, Mst1, Mst2, Sav1, Sav2) in mouse hearts 24, 48 and 72 hours post MI injury with control and miR-19a/19b mimic injection. Data are presented as mean ± SEM; $*P < 0.05$, relative to control. N=3 for MI-24h samples; N=4-5 for MI-48h samples, N=5-6 for MI-72h samples.

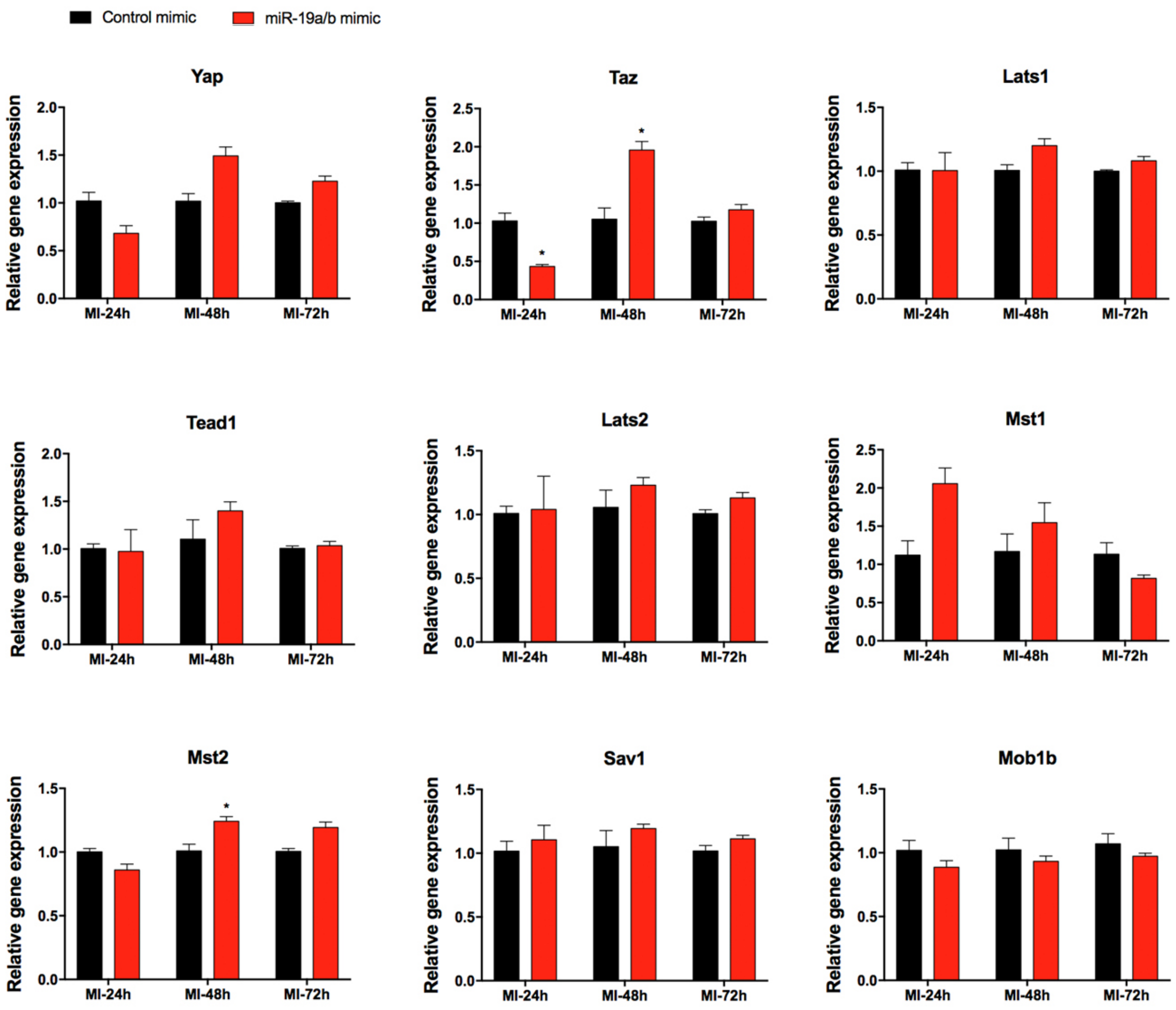

Supplementary Figure 4. Over expression of miR-19a/19b by AAV9 protected myocardium from apoptosis.

(A) Western blot analysis of apoptosis marker proteins Caspase-3 and Bim. (B) Quantification of Western blot analysis. N=3, Data are presented as mean ± SEM; *P < 0.05, relative to control.

A

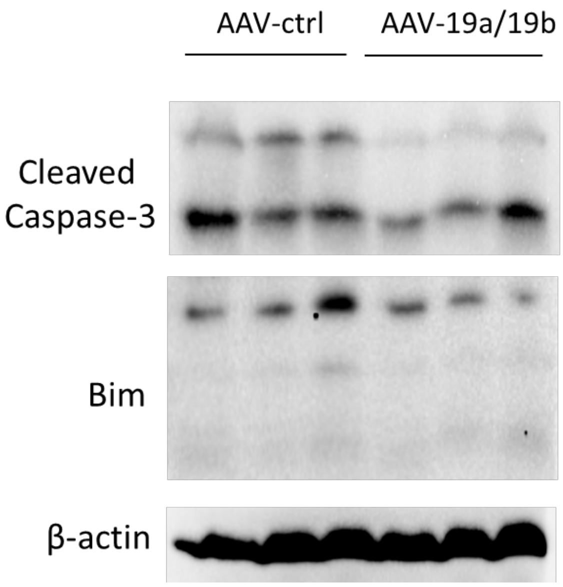

B

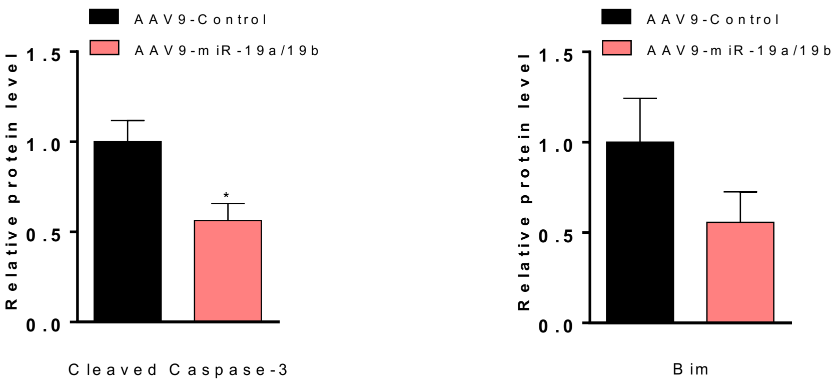

Supplementary Figure 5. Therapeutic potential of miR-19a/19b mimics via tail-vein RNAiMax delivered in protecting hearts from myocardial infarction injury. (A) Schematic of tail-vein injection of RNAiMax delivered miR-19a/19b or control mimics post MI injury. (B) miR-19a expression at 6 h after tail-vein injection post MI. (C-D) Echocardiography analyses of cardiac function in tail-vein injection of RNAiMax delivered miR-19a/19b or control mimics at different time points after MI. FS%, fractional shortening. LVID;s, LV internal dimension at end-systole. (D) Representative images of series of transverse heart sections of injection of miR-19a/19b or control mimic 14 weeks post MI injury. Sirius red/fast green collagen staining marks myocardium (green) and scar (red). (E) Quantification of the size of scar. N=3. Data are presented as mean ± SEM; *P < 0.05, ***P < 0.01, relative to control.

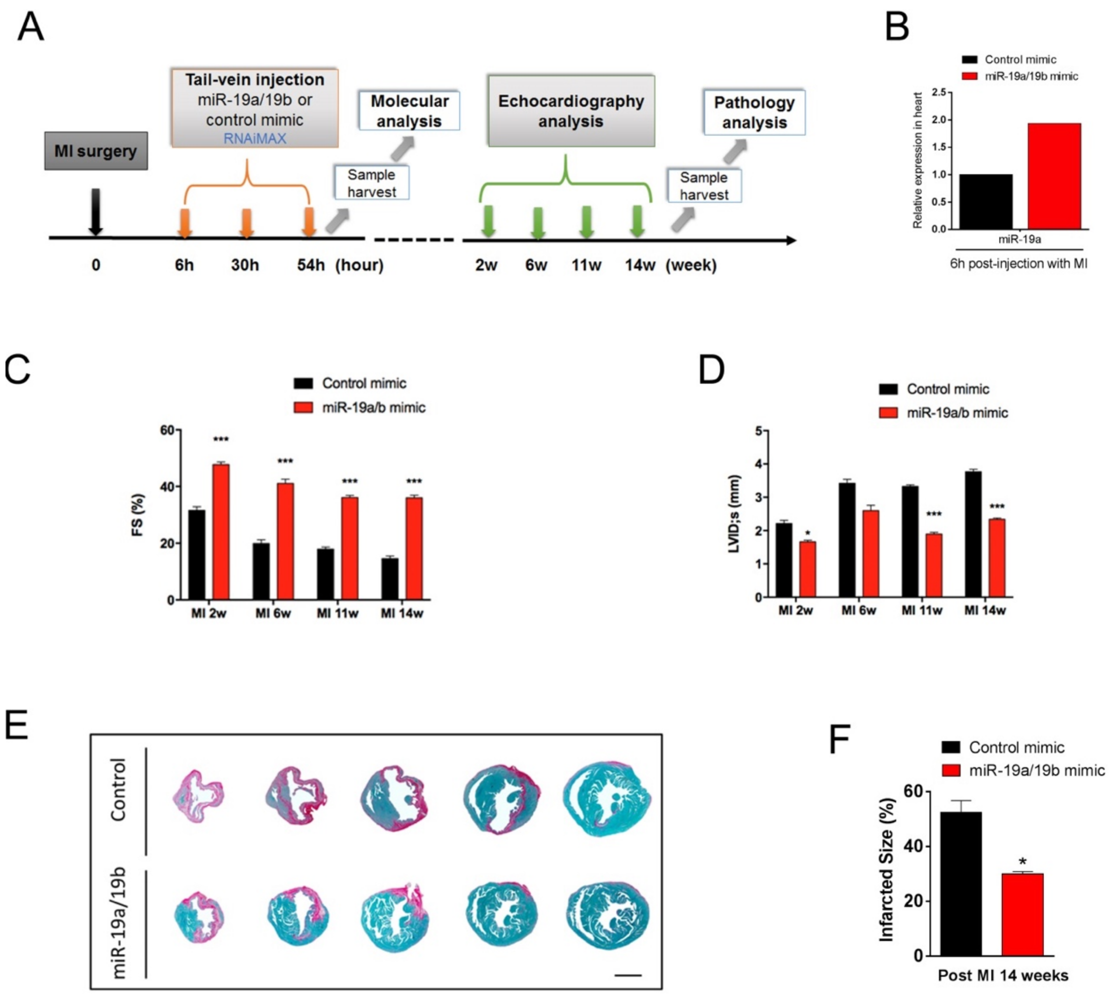

Supplement: Supplementary file 1 — Supplementary Information [file 41467_2019_9530_MOESM1_ESM.pdf]
